# Accurate and rapid prediction of tuberculosis drug resistance from genome sequence data using traditional machine learning algorithms and CNN

**DOI:** 10.1038/s41598-022-06449-4

**Published:** 2022-02-14

**Authors:** Xingyan Kuang, Fan Wang, Kyle M. Hernandez, Zhenyu Zhang, Robert L. Grossman

**Affiliations:** 1 Center for Translational Data Science, The University of Chicago, Chicago, IL 60615 USA; 2 Department of Medicine, The University of Chicago, Chicago, IL 60637 USA

**Keywords:** Genome informatics, Machine learning, Predictive medicine, Medical genetics, Mutation, Computational biology and bioinformatics, Genetics

## Abstract

Effective and timely antibiotic treatment depends on accurate and rapid in silico antimicrobial-resistant (AMR) predictions. Existing statistical rule-based *Mycobacterium tuberculosis* (MTB) drug resistance prediction methods using bacterial genomic sequencing data often achieve varying results: high accuracy on some antibiotics but relatively low accuracy on others. Traditional machine learning (ML) approaches have been applied to classify drug resistance for MTB and have shown more stable performance. However, there is no study that uses deep learning architecture like Convolutional Neural Network (CNN) on a large and diverse cohort of MTB samples for AMR prediction. We developed 24 binary classifiers of MTB drug resistance status across eight anti-MTB drugs and three different ML algorithms: logistic regression, random forest and 1D CNN using a training dataset of 10,575 MTB isolates collected from 16 countries across six continents, where an extended pan-genome reference was used for detecting genetic features. Our 1D CNN architecture was designed to integrate both sequential and non-sequential features. In terms of F1-scores, 1D CNN models are our best classifiers that are also more accurate and stable than the state-of-the-art rule-based tool Mykrobe predictor (81.1 to 93.8%, 93.7 to 96.2%, 93.1 to 94.8%, 95.9 to 97.2% and 97.1 to 98.2% for ethambutol, rifampicin, pyrazinamide, isoniazid and ofloxacin respectively). We applied filter-based feature selection to find AMR relevant features. All selected variant features are AMR-related ones in CARD database. 78.8% of them are also in the catalogue of MTB mutations that were recently identified as drug resistance-associated ones by WHO. To facilitate ML model development for AMR prediction, we packaged every step into an automated pipeline and shared the source code at https://github.com/KuangXY3/MTB-AMR-classification-CNN.

## Introduction

Antimicrobial resistance (AMR) is recognized as one of the greatest concerns for public health globally^1^. Previous work estimated that the deaths attributable to antimicrobial resistance might rise from the current estimate of 700,000 lives per year to ten million annually by 2050^2^. The prevalence of bacterial strains’ resistance to antibiotics has reduced the efficacy of antibiotics treatment dramatically^3^, which leads to the urgent need for antimicrobial susceptibility testing to guide the treatment of antibiotics for serious bacterial infections. The conventional culture-based methods have limitations including extended turnaround time for slow-growing bacteria such as Mycobacterium tuberculosis (MTB) and bias due to potential contamination. MTB remains the world’s most deadly infectious disease, with an estimated 1.5 million deaths in 2019^4^. The currently recommended treatment for drug-susceptible TB disease is a 6-month course of four first-line drugs: isoniazid (INH), rifampicin (RIF), ethambutol (EMB) and pyrazinamide (PZA)^5^. As resistance to first-line drugs has become more prevalent, second-line drugs were developed to treat first-line drug-resistant TB disease, which requires a course of second-line drugs for at least nine months and up to 20 months^4^. The emergence of drug-resistant TB continues to threaten global TB control efforts. The World Health Organization reported that nearly half a million people developed rifampicin-resistant TB (RR-TB), of which 78% had multidrug-resistant TB (MDR-TB) around the world in 2019^4^. There is an urgent need to rapidly identify drug sensitivity profiles of TB, given the fact that culture-based diagnostic tests are usually time-consuming.

To overcome these restrictions and identify antibiotic resistance more efficiently, researchers use conventional association rule methods to predict antimicrobial resistance^6^. These methods are based on the identification of variants associated with AMR from whole genome sequencing (WGS) data. The WGS data from clinical strains has been curated in dedicated databases including the Comprehensive Antibiotic Resistance Database (CARD)^7^ and the Pathosystems Resource Integration Center (PATRIC) ^8^.

Traditional machine learning (ML) algorithms, e.g., support vector machine (SVM), logistic regression (LR) and random forests (RF), have been compared with variant-based association rules for AMR prediction using WGS data of pathogen isolates in recent years^9,10^. Yang et al. developed and compared different traditional ML methods using a cohort of 1839 UK MTB isolates for the prediction of resistance on eight anti-TB drugs. Kouchaki et al. trained their models by using a dataset of over 13,402 isolates for more stable prediction on seen and unseen samples^10^. Three basic ML classifiers based on the feature space after dimension reduction and three ensemble learning methods were considered on this dataset. Another study conducted by Zhang et al. investigated deep learning strategy by using 2D Convolutional Neural Network (CNN) on whole-genome sequencing data of 149 MTB isolates for resistance classification on a less studied drug PZA^11^. Variants were called by aligning reads on a single reference genome H37Rv. Although ML, including deep learning, has been applied to the prediction of AMR, most studies used a limited number of isolates collected from a specific area, and all of them used single strain reference when detecting variants instead of using pan-genome reference^12,13^, which could result in poor mapping and variant calling quality in new strains. The use of a pan-genome reference can decrease errors in the mapping and variant detection process, especially for more diverged strains.

Here, we present our study of MTB drug resistance classification using traditional ML methods (LR and RF) and a deep neural network architecture of 1D CNN on a large and diverse dataset of MTB isolates. To compare the performance of our ML classifiers with a state-of-the-art statistical modeling method Mykrobe predictor, we evaluated the accuracy of Mykrobe predictor on the same dataset^14^. Mykrobe predictor uses a De Bruijn graph representation of bacterial diversity to identify species and resistance profiles of clinical isolates for Staphylococcus aureus and Mycobacterium tuberculosis. We used a dataset of 10,575 MTB isolates^15^, which is imbalanced with more susceptible isolates than resistant ones for all four first-line drugs mentioned above and four second-line drugs: amikacin (AMK), capreomycin (CM), kanamycin (KM) and ofloxacin (OFX). To reduce computation, we performed feature selection first to reduce the dimensions of input data and applied multi-input 1D CNN. Instead of using a single strain reference, we used all references from CARD database^16^, even including references of other bacteria to build reference clusters as a pan-genome reference. Sequencing reads were then aligned to these reference clusters for variant detection. The results showed that our best ML classifiers outperformed the state-of-the-art rule-based method Mykrobe predictor, especially for EMB resistance, and showed more stable accuracy to all the four first-line drugs. Although our basic 1D CNN architecture didn’t significantly outperform our traditional ML methods LR and RF, there are potential ways to optimize it in the future, e.g., hyperparameter tuning.

## Methods

### Data collection

**Figure 1:**
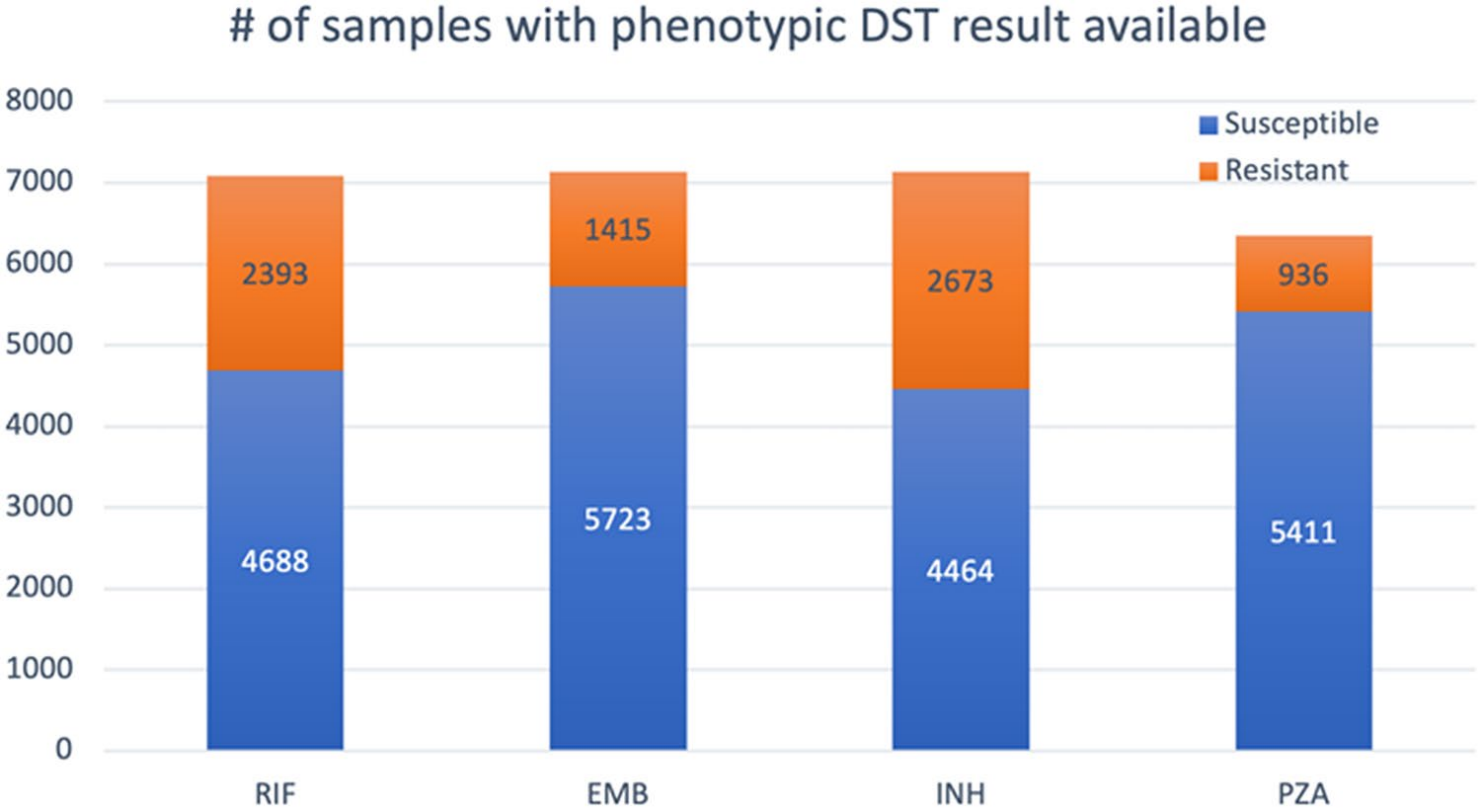
Phenotypic overview of the MTB isolates. This bar chart shows numbers of susceptible and resistant isolates with DST results available for each of the four first-line drugs.

To prepare the training data and labels, we downloaded the whole-genome sequencing (WGS) data for 10,575 MTB isolates from the sequence read archive (SRA) database^17^ and obtained corresponding lineage and phenotypic drug susceptibility test (DST) data from CRyPTIC Consortium and the 100,000 Genomes project in an excel file, which is also available in the supplementary of their publication^15^. The phenotypic DST results for the drugs were used as labels when training and evaluating our ML models. All the data were collected and shared by the CRyPTIC Consortium and the 100,000 Genomes Project^15^. Like the datasets used by previous studies, this dataset is imbalanced in that most isolates are susceptible, and the minority of them are resistant for all the four first-line drugs (Fig. 1) and four second-line drugs. The numbers of isolate samples with phenotypic DST results available are 7138, 7137, 6347 and 7081 for EMB, INH, PZA and RIF, respectively. There are 6291 shared isolates among the four sample sets. In addition, 6820 out of the 10,575 isolates have phenotypic DST result available for each of the four second-line drugs.

### Genetic feature extraction

To detect the potential genetic features that could contribute to MTB drug resistance classification, we used a command-line tool called ARIBA^18^. ARIBA is a very rapid, flexible and accurate AMR genotyping tool that generates detailed and customizable outputs from which we extracted genetic features. First, we downloaded all reference data from CARD, which included not only references from different MTB strains but also from other bacteria (e.g., *Staphylococcus aureus*). Secondly, we clustered reference sequences based on their similarity. Then we used this collection of reference clusters as our pan-genome reference and aligned read pairs of an isolate to them. For each cluster that had reads mapped, we ran local assemblies, found the closest reference, and identified variants. After running these steps, ARIBA generated files including a summary file for alignment quality, a report file containing information of detected variants and AMR-associated genes, and a read depth file. For each cluster, the read depth file provides counts of the four DNA bases on each locus of the closest reference where reads were mapped.

Next, we filtered out low-quality mappings that did not pass the ‘match’ criteria defined in ARIBA’s GitHub wiki^18^. From these high-quality mappings, we collected novel variants in coding regions, well-studied resistance-causing variants and AMR-associated gene presences that were detected from at least one out of the 10,575 isolates as 263 genetic features. In addition, we included indicator variables for each of the 19 lineages into our feature vector resulting in a total of 282 features.

### Traditional ML methods

We applied two traditional ML algorithms, RF and LR, on the sample sets labeled with phenotypic DST results (see “Data collection” section) to train MTB AMR classifiers for the eight drugs (first-line and second-line), where the feature vector for each sample consists of the 282 features mentioned in “Genetic feature extraction” section.

RF is an ensemble method and made up of tens or hundreds of estimators (decision trees) to compress overfitting^19,20^. A final prediction is an average or majority vote of the predictions of all trees. It is often used when there are large training datasets and a large number of input features. Moreover, RF is good at dealing with imbalanced data by using class weighting. Here we trained each RF classifier with 1000 estimators.

LR is a popular regression technique for modeling binary dependent variable^21^. By using a sigmoid function (logit), linear regression is transformed into logistic regression so that the prediction range is [0, 1] for outputting probabilities. Then, LR model is fitted using maximum likelihood estimation. During the training process, we applied L1 regularization on LR models for feature selection and to prevent overfitting^22^.

### Feature selection and 1D CNN models

CNN is a class of deep neural networks that takes multi-dimensional data as input^23^. When we say CNN, generally, we refer to a 2-dimensional CNN, which is often used for image classification. However, there are two other types of CNN used in practice: 1-dimensional and 3-dimensional CNNs. Conv1D is generally used for time-series data where the kernel moves on one dimension and the input and output data are 2-dimensional. Conv2d and 3D kernels move on two dimensions and three dimensions, respectively.

**Figure 2:**
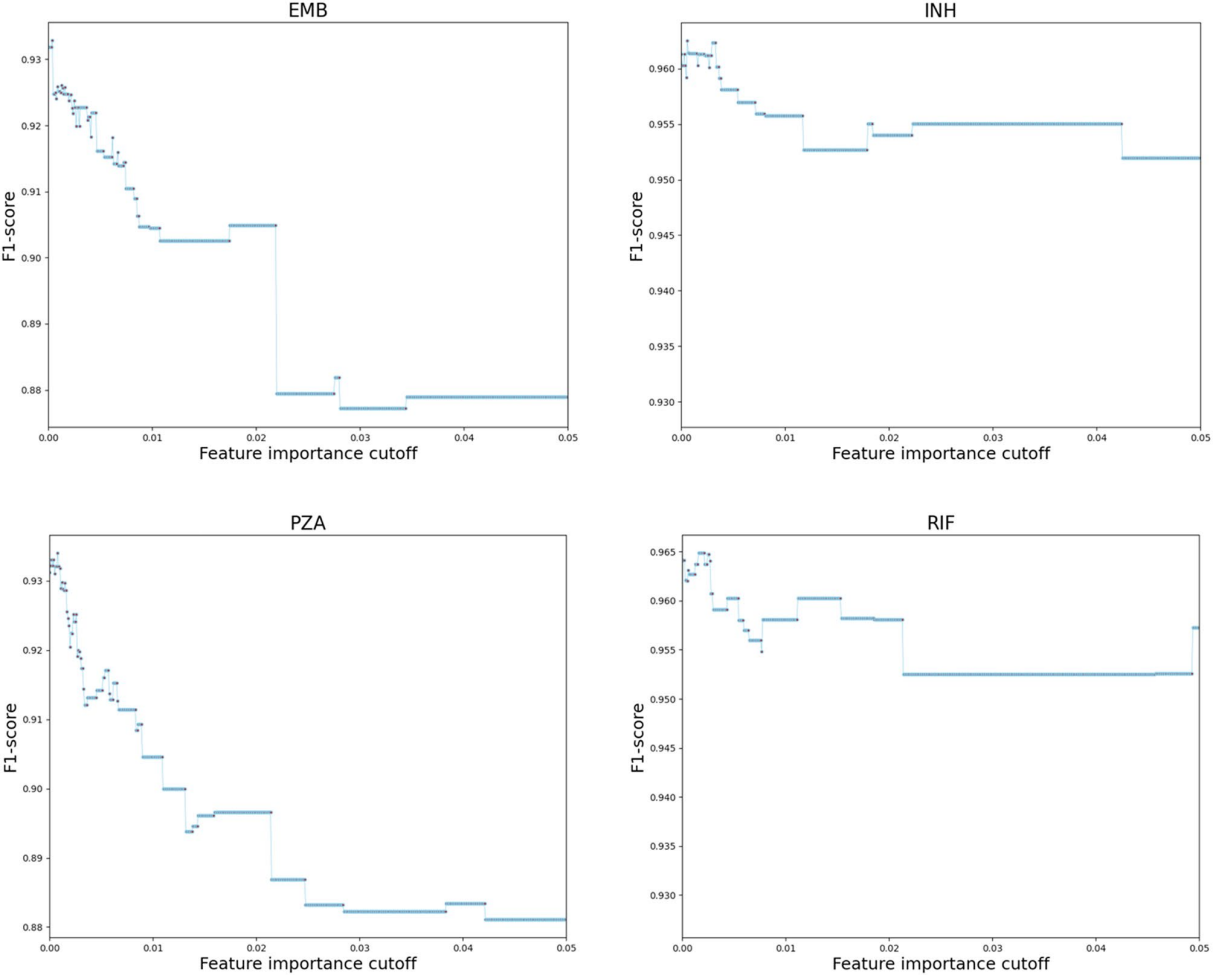
Feature importance cutoff tuning. For the four first-line drugs, when the cutoff increases, the F1-score quickly increases to its maximum and then continues to decrease. The cutoffs maximized F1-scores are 0.0004 (EMB), 0.0006 (INH), 0.0008 (PZA) and 0.0016 (RIF).

Because deep learning algorithms require substantial computational power, we performed feature selection to only keep relevant features as input for deep learning algorithms. First, we randomly selected 80 percent of samples to calculate the importance of each feature by using the scikit-learn RF feature importance function that averages the impurity decrease from each feature across the trees to determine the final importance of each variable^24^. Then, we tuned the feature importance cutoff to find the one that maximizes the F1-score of an RF model trained on the remaining 20 percent of samples. For each of the eight drugs, features were selected when their feature importance scores were bigger than the optimal cutoff. The tuning processes for first-line drugs are visualized in Fig. 2.

**Figure 3:**
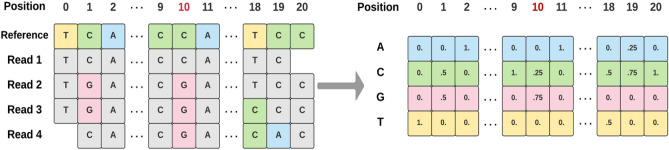
Conversion of raw base counts at each locus of a 21-base reference window into normalized base counts as Conv1D input of each selected SNP feature. The raw base counts were derived from reference-reads alignment, as shown on the left of this figure. The center of the window is the locus of a selected SNP feature. The normalized base counts at each locus are the percentage of the four DNA bases (ACGT), respectively.

**Figure 4:**
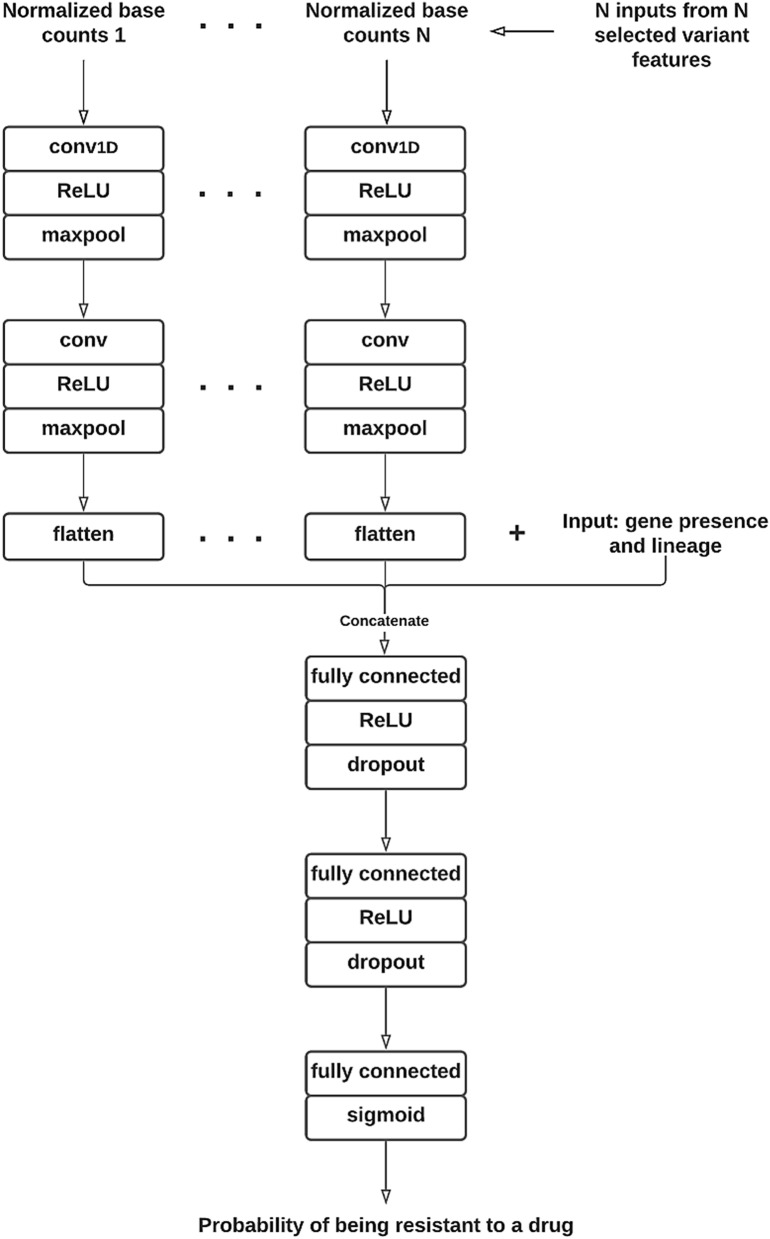
Flowchart of our 1D CNN architecture.

After the relevant features were selected, we designed and built a multi-input CNN architecture with TensorFlow Keras^25^ that took N inputs of 4 × 21 matrices representing N selected SNP features into the first layer. Each 4 × 21 matrix consists of normalized DNA base counts for each locus within a 21-base reference sequence window centered on the focal SNP (Fig. 3). We generated normalized counts based on the raw base counts extracted from the read depth file mentioned in “Genetic feature extraction” section. Our convolutional architecture starts with two 1D convolutional layers followed by a flattening layer for each SNP input. Then, it concatenates the N flattening layers with the inputs of AMR-associated gene presence and lineage features. Finally, we added three fully connected layers to complete the deep neural network architecture (Fig. 4). It smoothly integrates sequential and non-sequential features.

## Results

### Isolate identification and DST phenotype

**Figure 5:**
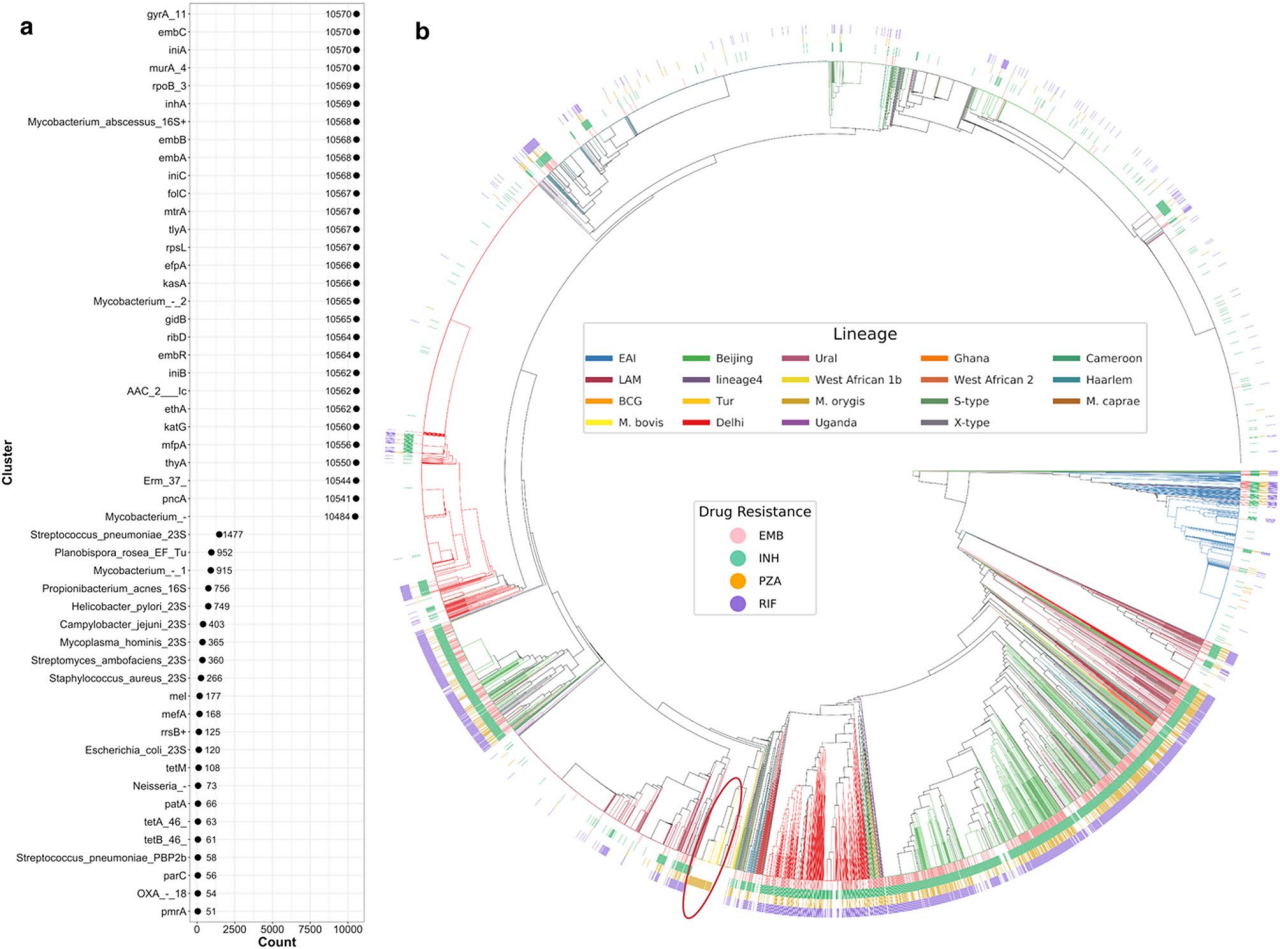
Visualization of isolate identification from detected genetic information, lineage and DST. (**a**) Numbers of matched isolates on each reference cluster that has more than 50 isolates matched on. (**b**) Circular phylogenetic tree with lineage and phenotypic DST data annotation. Resistance co-occurrence frequently happens according to this visualization. However, one exception is the highlighted branch of isolates resistant to PZA but susceptible to the other three drugs. This figure was drawn by using plotTree^26^.

To explore genetic information obtained by running the ARIBA steps listed in “Genetic feature extraction” section, we calculated the numbers of isolates matched on different reference clusters (Fig. 5a) and generated a circular phylogenetic tree with lineage and phenotypic DST data annotations (Fig. 5b).

Figure 5a shows that reads from most isolates were mapped on MTB reference clusters by using the ‘match’ criteria, while only a small portion of isolates were matched to reference clusters of other bacteria (clusters below ‘Mycobacterium_-’ in Fig. 5a, e.g., *Staphylococcus aureus*). By using a pan-genome reference, we can get more reliable alignments to detect variants more accurately^27^. The 6291 isolates with phenotypic DST results available for the four first-line drugs were clustered into a phylogenetic tree (Fig. 5b). The inner circle is a phylogenetic tree based on the genetic information (reference cluster matches and known variants) detected by ARIBA, where leaves (isolates) were colored according to their lineage information. Here, isolates of the same lineage clustered together, providing confidence in the quality of isolate identification from genetic information. The outer circles show phenotypic DST of resistance or susceptibility to the four drugs for each isolate. Taken together, there are clear patterns and relationships among lineages, AMR phenotype, and genetic data.

### Selected features for 1D CNN

After we performed the feature selection, the top 42 (RIF), 68 (INH), 113 (PZA) and 125 (EMB) drug-specific features were collected. Across these four sets, there were 42 shared features, indicating that the 42 features selected for RIF resistance prediction are also relevant to AMR classification of the other three drugs (Additional file 1: Fig. S1). We also ran the same feature selection procedure on second-line drugs: amikacin (AMK), capreomycin (CM), kanamycin (KM) and ofloxacin (OFX). For each of the eight (first- and second-line) drugs, all selected variant features are known AMR-associated variants from the current version of CARD database (Nov. 2021). We compared our selected variants with the AMR-associated mutations in MTB that were recently published by WHO^28^. We list all selected variants and highlight the ones overlapping with WHO’s AMR-associated MTB mutations in Additional file 1: Table S1. Overall, we have 78.8% selected variants that are also in WHO’s list.

### Training and evaluation

We performed tenfold cross-validation to train and test 24 binary classifiers of AMR status across the eight (first- and second-line) drugs and three different ML algorithms: LR, RF and customized 1D CNN. The four datasets described in “Data collection” section were used to train and test our first-line drug-specific models. In addition, we collected training data from the 6820 out of the 10,575 isolates, trained and tested ML AMR classifiers for second-line drugs by applying the same steps as for first-line drugs. The second-line drugs are listed in last section “Selected features for 1D CNN”. To compare our models with a rule-based method, we also tested the state-of-the-art AMR prediction tool Mykrobe predictor on the same sample sets used for the eight TB drugs, respectively. The precision, sensitivity, specificity, accuracy, F1-score and G-mean were calculated to evaluate the different methods (Table 1).

$$\mathrm{Precision}=\frac{\mathrm{TP}}{\mathrm{TP}+\mathrm{FP}} ,\mathrm{ Sensitivity}=\frac{\mathrm{TP}}{\mathrm{TP}+\mathrm{FN}},$$

$$\mathrm{Specificity}=\frac{\mathrm{TN}}{\mathrm{TN}+\mathrm{FP}} ,\mathrm{ Accuracy}=\frac{\mathrm{TP}+\mathrm{TN}}{\mathrm{TP}+\mathrm{FP}+\mathrm{TN}+\mathrm{FN}},$$

$$\mathrm{F}1-\mathrm{score}=\frac{2\times \mathrm{Precision}\times \mathrm{Sensitivity}}{\mathrm{Precision}+\mathrm{Sensitivity}},\mathrm{ G}-\mathrm{mean}=\sqrt{\mathrm{Sensitivity}\times \mathrm{Specificity}},$$

where TP, TN, FP and FN are true positive, true negative, false positive and false negative, respectively. We used the default probability threshold of 0.5 to decide whether it is susceptible or resistant for all our ML models; however, the performance of our models could be improved in the future by tuning this hyperparameter.

**Table 1:** Evaluation of AMR classifiers for first-line and second-line anti-TB drugs (our ML methods VS the rule-based one Mykrobe predictor).

Methods	Precision (%)	Sensitivity (%)	Specificity (%)	Accuracy (%)	F1 (%)	G-mean (%)
**First-line**						
**INH**						
RF	95.2	98.7	91.7	96.1	97.0	95.1
LR	94.3	99.2	90.0	95.7	96.7	94.5
CNN	95.5	99.0	90.5	96.2	97.2	94.7
Mykrobe	92.9	99.2	95.3	96.2	95.9	97.2
**PZA**						
RF	92.3	96.4	53.6	90.1	94.3	71.9
LR	93.0	95.5	58.0	90.0	94.2	74.4
CNN	93.2	96.5	56.1	90.5	94.8	73.6
Mykrobe	91.1	95.1	60.9	87.3	93.1	76.1
**RIF**						
RF	94.3	97.0	88.6	94.1	95.6	92.7
LR	93.5	98.3	86.6	94.3	95.8	92.3
CNN	94.4	98.1	87.5	94.6	96.2	92.7
Mykrobe	92.4	95.0	92.3	92.5	93.7	93.6
**EMB**						
RF	92.9	94.4	70.8	89.7	93.6	81.8
LR	93.1	93.4	72.1	89.2	93.3	82.1
CNN	93.1	94.5	71.7	90.0	93.8	82.3
Mykrobe	92.2	72.4	85.3	76.3	81.1	78.6
**Second-line**						
**AMK**						
RF	99.1	99.7	80.9	98.9	99.4	89.8
LR	99.2	99.9	82.8	99.2	99.5	91.0
CNN	99.2	100	82.9	99.2	99.6	91.1
Mykrobe	99.1	100	81.9	99.2	99.5	90.5
**CM**						
RF	96.0	98.7	48.0	94.9	97.3	68.8
LR	96.1	99.9	49.0	96.1	98.0	70.0
CNN	96.1	99.9	49.0	96.1	98.0	70.0
Mykrobe	96.0	99.9	47.8	96.1	97.8	69.1
**KM**						
RF	94.0	93.9	59.0	89.4	93.9	74.4
LR	94.5	95.1	63.0	91.0	94.8	77.4
CNN	93.0	98.4	49.8	92.1	95.6	70.0
Mykrobe	90.9	99.5	31.9	91.0	95.0	56.4
**OFX**						
RF	97.5	98.5	64.5	96.4	98.0	79.7
LR	97.6	99.1	65.2	96.9	98.3	80.4
CNN	97.5	98.9	63.4	96.6	98.2	79.2
Mykrobe	98.5	95.9	78.1	94.8	97.1	86.6

We calculated different metrics to measure the performances of the four approaches (Table 1). The F1-score is the harmonic mean of precision and sensitivity and balances precision and sensitivity equally. Since the F1-score does not consider True Negatives (TN) we also included the geometric mean of sensitivity and specificity (G-mean) as an additional metric. However, in cases of imbalanced classes, the interpretability of these various metrics starts to break down. Although it is imperfect, we focus on F1-scores because the equal balance between precision and recall is relevant for our interpretation and is important for reducing bias in imbalanced datasets. In terms of F1-score, our three ML methods outperformed the rule-based method Mykrobe predictor for all the four first-line drugs and one of the second-line drugs, while the 1D CNN classifier achieved the highest scores overall.

In their manuscript, the Mykrobe predictor authors stated that the sensitivity of their MTB drug resistance prediction was low, potentially because their graph-based association rule had limited understanding of the underlying genetic mechanisms. We confirmed their observation when testing Mykrobe predictor on our datasets. As shown in Table 1, for EBM, our best model greatly improved the sensitivity from 72.4 to 94.5%, suggesting that our 1D CNN models can detect more complex or subtle genetic mechanisms.

## Discussion

According to our tenfold cross-validation, our best ML classifiers showed a substantial increase in the F1-score for all the four first-line drugs and one second-line drug when compared to the prediction from the state-of-the-art rule-based method Mykrobe predictor. Our 1D CNN architecture only slightly outperformed the traditional ML methods LR and RF, although it requires more intensive computing resources during the training process. To reduce the computing resource requirements, we performed feature selection to remove irrelevant features before training 1D CNN models. For each drug, all selected variant features are known variants based on the current version of CARD. In this study, a special 1D CNN architecture was built to fit our data structure of mixed-type of data (sequential and non-sequential). As our first-stage study for MTB AMR classification, we didn’t perform hyperparameter optimization, but it is a potential way to improve our models in the future. In addition, we can include novel variants on non-coding regions and larger variants (e.g. indel) as additional features and try the computationally expensive wrapper-type feature selection algorithms (e.g., recursive feature elimination^29^) to compare with the filter-based one used in this study^30^. Because ARIBA was not focusing on detection of low-frequency variants in NGS data and low-frequency variants are also associated to AMR classification, we could add low-frequency variants as additional features for training our ML model by using specific SNP detection tool like binoSNP^31^ in our future work.

The large and diverse dataset of mycobacterium isolates used in our study ensures more generally trained models to predict future samples more accurately, presumably because it can better manage overfitting than regularization on a less diverse dataset. It is important to note that TB drug resistance in Mycobacterium tuberculosis is not known to involve plasmids. To extend our model into bacteria where plasmids have a role in resistance, there would need to make sure the reference database for generating reference clusters using ARIBA contains complete plasmid sequences like CARD that we used in this study. In this way, the additional plasmid features could be easily integrated into the models as presented here.

Although we focused on the F1-score as our metric of performance because it balances precision with recall, it does receive criticism because it ignores True Negatives (TN). In many clinical settings both specificity and sensitivity have critical impacts on patients and the care they receive. We also presented the G-score which is simply the geometric mean of sensitivity and specificity; however, interpretation may be biased in cases where there is an imbalance of classes (e.g., number of resistant versus non-resistant isolates). When focusing on this metric, there is more variability in performance outcomes between the rule-based and the ML methods presented here. Regardless, across all these methods substantial gains in specificity are possible and should be a focus of future work in this area.

Finally, we automated the whole process, from data collection to model training and evaluation, into a flexible pipeline that can be easily updated with new strains or train AMR prediction models of different antibiotics for other bacteria (Additional file 1: Fig. S2 for an overview of the pipeline). Given the availability of WGS data and lineage information for MTB, our ML models can classify MTB resistance against the eight anti-TB drugs with relatively high accuracy requiring only the computational resources of a standard laptop.

## Conclusions

AMR infection is one of the major threats to human health. In silico methods are effective to predict drug resistance and a reliable alternative to in vitro assay that is much slower and more expensive. Statistical association rule and ML are two main types of in silico approaches. We developed ML models for first-line TB drug resistance classification on a large and diverse MTB isolate cohort to compare to a statistical rule-based method. The result shows our ML models are more accurate and stable for TB drug resistance prediction across the four first-line drugs than the rule-based method Mykrobe predictor. We designed and developed a customized 1D CNN architecture to adapt and combine sequential and non-sequential features. Even though our deep CNN models haven’t taken advantage of any optimization strategies (e.g., hyperparameter tuning), our CNN architecture slightly outperformed the other two traditional ML algorithms. As a result of variant analysis, 78.8% of variant features selected for our CNN model training are also identified as TB drug resistance-associated ones by WHO.

## Supplementary Information

Supplementary Information.

## Data Availability

The WGS data of the MTB cohort analyzed in this study are available in SRA database. Code of the ML model development pipeline written for this study is available at https://github.com/KuangXY3/MTB-AMR-classification-CNN.

## Abbreviations

MTB: *Mycobacterium tuberculosis*
AMR: Antimicrobial-resistant
ML: Machine learning
LR: Logistic regression
RF: Radom forest
CNN: Convolutional Neural Network
INH: Isoniazid
RIF: Rifampicin
EMB: Ethambutol
PZA: Pyrazinamide
MDR-TB: Multidrug-resistant
CARD: Comprehensive Antibiotic Resistance Database
PATRIC: Pathosystems Resource Integration Center
SVM: Support vector machine
WGS: Whole-genome sequencing
SRA: Sequence read archive
DST: Drug susceptibility test
TP: True positive
TN: True negative
FP: False positive
FN: False negative
