# Supplementary material for: Accurate and rapid prediction of tuberculosis drug resistance from genome sequence data using traditional machine learning algorithms and CNN
Source: Sci Rep. 2022 Feb 14;12:2427. doi: 10.1038/s41598-022-06449-4 (PMC8844416; doi:10.1038/s41598-022-06449-4)
Supplement: Supplementary file 1 — Supplementary Information. [file 41598_2022_6449_MOESM1_ESM.pdf]

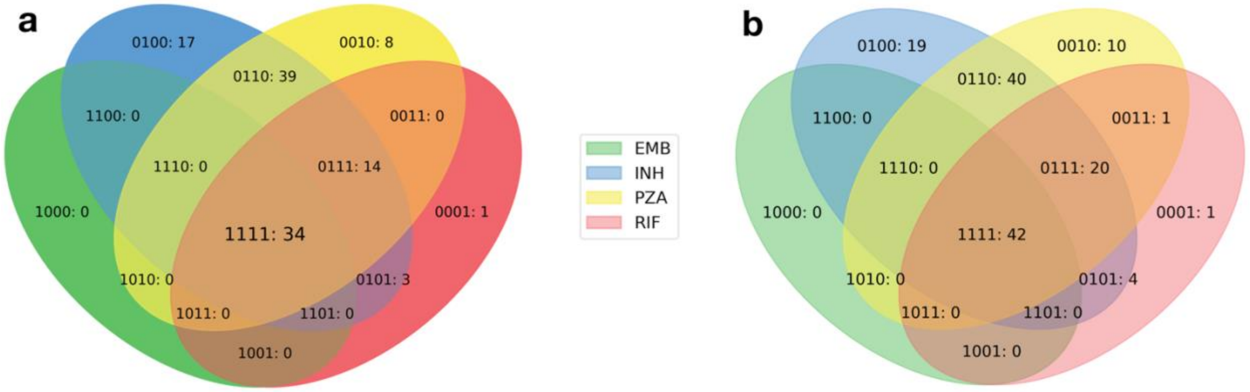

Fig. S1. Venn diagrams showing how the four sets of selected features overlap with each other. These features were selected for training 1D CNN models for the four first-line drugs. **a** Only variant features. **b** All types of features. These Venn diagrams were generated by using a python package called pyvenn (https://github.com/tctianchi/pyvenn)

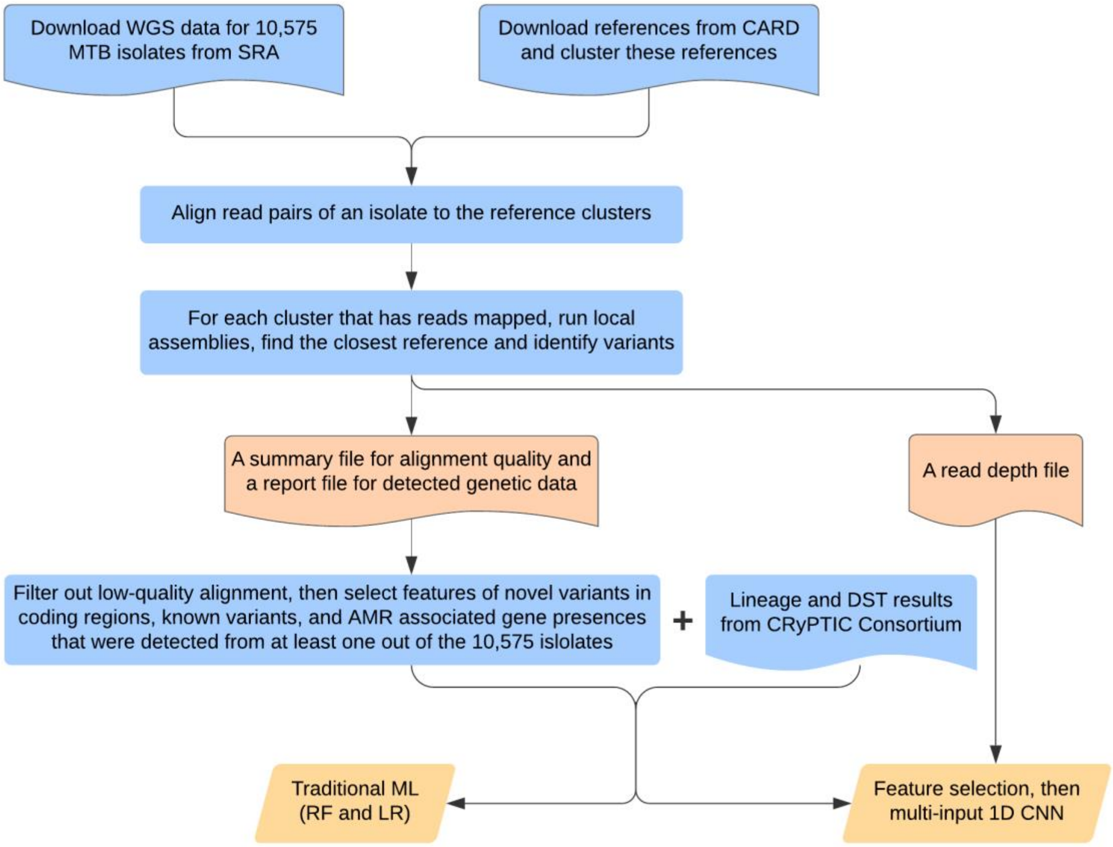

Fig. S2. Flowchart of our ML model development for MTB drug resistance classification.

Table S1. Selected variant features for 1D CNN model training of the eight (first-line and second-line) anti-TB drugs. Variants that are also in the list of AMR-associated TB mutations recently published by WHO are highlighted in bold.

Drugs	Selected variants	Covered by WHO (%)
	**First-line**	
RIF	**embB.D1024N**<br>**embB.G406A**<br>**embB.G406D**<br>**embB.G406S**<br>**embB.M306I**<br>**embB.M306L**<br>**embB.M306V**<br>**embB.Q497K**	82.35%

	**embB.Q497R**<br>**embC.R738Q**<br>**embC.T270I**<br>**embC.V981L**<br>**gyrA.A90V**<br>**gyrA.D94A**<br>**gyrA.D94G**<br>**gyrA.D94N**<br>**gyrA.D94Y**<br>**gyrA.S91P**<br>**inhA.I194T**<br>**inhA.I21T**<br>**inhA.S94A**<br>**katG.R463L**<br>**katG.S315N**<br>**katG.S315T**<br>**rpoB.S450L**<br>**rpsL.K43R**<br>**rpsL.K88R**<br>**Mycobacterium_tuberculosis_16S.A1401G**<br>katG.A234G<br>katG.G300W<br>katG.A431V<br>kasA.G269S<br>Mycobacterium_avium_23S.A2274G<br>gidB.A134E	
EMB	**embA.P913S**<br>**embB.D1024N**<br>**embB.D328G**<br>**embB.D328Y**<br>**embB.E378A**<br>**embB.G406A**<br>**embB.G406C**<br>**embB.G406D**<br>**embB.G406S**<br>**embB.M306I**<br>**embB.M306L**<br>**embB.M306V**<br>**embB.Q497K**<br>**embB.Q497R**<br>**embB.S297A**<br>**embB.Y319C**<br>**embB.Y334H**<br>**embC.N394D**<br>**embC.R738Q**<br>**embC.T270I**	79.44 %

	**embC.V981L**<br>**embR.C110Y**<br>**ethA.A381P**<br>**gyrA.A90V**<br>**gyrA.D89G**<br>**gyrA.D89N**<br>**gyrA.D94A**<br>**gyrA.D94G**<br>**gyrA.D94N**<br>**gyrA.D94Y**<br>**gyrA.G88A**<br>**gyrA.G88C**<br>**gyrA.S91P**<br>**inhA.I194T**<br>**inhA.I21T**<br>**inhA.I21V**<br>**inhA.S94A**<br>**katG.Q127P**<br>**katG.R463L**<br>**katG.S140N**<br>**katG.S315G**<br>**katG.S315I**<br>**katG.S315N**<br>**katG.S315T**<br>**katG.Y337C**<br>**ndh.R268H**<br>**ndh.V18A**<br>**pncA.A146T**<br>**pncA.C14R**<br>**pncA.D12A**<br>**pncA.D12N**<br>**pncA.D49A**<br>**pncA.F58L**<br>**pncA.G132S**<br>**pncA.G97S**<br>**pncA.H82R**<br>**pncA.L172P**<br>**pncA.L27P**<br>**pncA.L4W**<br>**pncA.L85P**<br>**pncA.L85R**<br>**pncA.M175V**<br>**pncA.P54L**<br>**pncA.Q10P**<br>**pncA.Q10R**<br>**pncA.Q141P**	

	**pncA.R154G**<br>**pncA.S67P**<br>**pncA.T135P**<br>**pncA.T160P**<br>**pncA.T47A**<br>**pncA.T76P**<br>**pncA.V139A**<br>**pncA.V155G**<br>**pncA.V7G**<br>**pncA.V9G**<br>**pncA.W68G**<br>**pncA.W68R**<br>**pncA.Y103H**<br>**rpoB.S450L**<br>**rpsL.K43R**<br>**rpsL.K88Q**<br>**rpsL.K88R**<br>**Mycobacterium_tuberculosis_16S.A1401G**<br>**tlyA.N236K**<br>katG.A234G<br>katG.G300W<br>katG.A431V<br>rpoB.L511R<br>rpoB.D516G<br>rpoB.H526T<br>kasA.G312S<br>kasA.G269S<br>thyA.T202A<br>gidB.S70R<br>Mycobacterium_avium_23S.A2274G<br>folC.A420V<br>iniA.S501W<br>gidB.P75S<br>gidB.A134E<br>folC.S150G<br>gidB.A138E<br>folC.I43S<br>thyA.H207R<br>gidB.H48Y<br>folC.I43A<br>katG.P131Q	
INH	**embA.P913S**<br>**embB.D1024N**<br>**embB.D328Y**<br>**embB.E378A**<br>**embB.G406A**	84.62%

	**embB.G406C**<br>**embB.G406D**<br>**embB.G406S**<br>**embB.M306I**<br>**embB.M306L**<br>**embB.M306V**<br>**embB.Q497K**<br>**embB.Q497R**<br>**embC.N394D**<br>**embC.R738Q**<br>**embC.T270I**<br>**embC.V981L**<br>**embR.C110Y**<br>**ethA.A381P**<br>**gyrA.A90V**<br>**gyrA.D94A**<br>**gyrA.D94G**<br>**gyrA.D94N**<br>**gyrA.D94Y**<br>**gyrA.G88A**<br>**gyrA.S91P**<br>**inhA.I194T**<br>**inhA.I21T**<br>**inhA.I21V**<br>**inhA.S94A**<br>**katG.G279D**<br>**katG.Q127P**<br>**katG.R463L**<br>**katG.S315G**<br>**katG.S315I**<br>**katG.S315N**<br>**katG.S315T**<br>**ndh.R268H**<br>**ndh.V18A**<br>**pncA.H82R**<br>**rpoB.S450L**<br>**rpsL.K43R**<br>**rpsL.K88R**<br>**Mycobacterium_tuberculosis_16S.A1401G**<br>katG.A234G<br>katG.G300W<br>katG.A431V<br>kasA.G269S<br>thyA.T202A<br>Mycobacterium_avium_23S.A2274G<br>gidB.A134E	

	thyA.H207R	
PZA	**embA.P913S**<br>**embB.D1024N**<br>**embB.D328Y**<br>**embB.E378A**<br>**embB.G406A**<br>**embB.G406C**<br>**embB.G406D**<br>**embB.G406S**<br>**embB.M306I**<br>**embB.M306L**<br>**embB.M306V**<br>**embB.Q497K**<br>**embB.Q497R**<br>**embB.S297A**<br>**embB.Y319C**<br>**embB.Y334H**<br>**embC.N394D**<br>**embC.R738Q**<br>**embC.T270I**<br>**embC.V981L**<br>**embR.C110Y**<br>**ethA.A381P**<br>**ethA.D58A**<br>**gyrA.A90V**<br>**gyrA.D89G**<br>**gyrA.D94A**<br>**gyrA.D94G**<br>**gyrA.D94N**<br>**gyrA.D94Y**<br>**gyrA.G88A**<br>**gyrA.G88C**<br>**gyrA.S91P**<br>**inhA.I194T**<br>**inhA.I21T**<br>**inhA.I21V**<br>**inhA.S94A**<br>**katG.R463L**<br>**katG.S315N**<br>**katG.S315T**<br>**ndh.R268H**<br>**ndh.V18A**<br>**pncA.A134V**<br>**pncA.D12A**	82.11%

	**pncA.D12N**<br>**pncA.D49A**<br>**pncA.F58L**<br>**pncA.G132S**<br>**pncA.H51Q**<br>**pncA.H57D**<br>**pncA.H82R**<br>**pncA.L159R**<br>**pncA.L172P**<br>**pncA.L27P**<br>**pncA.L4W**<br>**pncA.L85P**<br>**pncA.L85R**<br>**pncA.M175V**<br>**pncA.P54L**<br>**pncA.Q10P**<br>**pncA.Q10R**<br>**pncA.Q141P**<br>**pncA.S67P**<br>**pncA.T135P**<br>**pncA.T160P**<br>**pncA.T76P**<br>**pncA.V139A**<br>**pncA.V139L**<br>**pncA.V7G**<br>**pncA.V9G**<br>**pncA.W119R**<br>**pncA.W68G**<br>**pncA.W68R**<br>**rpoB.S450L**<br>**rpsL.K43R**<br>**rpsL.K88Q**<br>**rpsL.K88R**<br>**Mycobacterium_tuberculosis_16S.A1401G**<br><br>**tlyA.N236K**<br>katG.A234G<br>katG.G300W<br>katG.A431V<br>kasA.G312S<br>kasA.G269S<br>thyA.T202A<br>gidB.S70R<br>Mycobacterium_avium_23S.A2274G<br>folC.A420V<br>iniA.S501W	

	folC.I43T<br>gidB.A134E<br>folC.S150G<br>gidB.A138E<br>folC.I43S<br>thyA.H207R<br>folC.I43A	
**Second-line**		
AMK	**katG.S315T**<br>**rpoB.S450L**<br>**rpsL.K43R**<br>**Mycobacterium_tuberculosis_16S.A1401G**<br>**embB.M306V**	100.00%
CM	**Mycobacterium_tuberculosis_16S.A1401G**	100.00%
KM	**embA.P913S**<br>**embB.D1024N**<br>**embB.E378A**<br>**embB.G406D**<br>**embB.G406S**<br>**embB.M306I**<br>**embB.M306V**<br>**embB.Q497R**<br>**embC.N394D**<br>**embC.R738Q**<br>**embC.T270I**<br>**embC.V981L**<br>**embR.C110Y**<br>**gyrA.A90V**<br>**gyrA.D94G**<br>**gyrA.D94N**<br>**gyrA.D94Y**<br>**gyrA.S91P**<br>**katG.S315T**<br>**ndh.V18A**<br>**rpoB.S450L**<br>**rpsL.K43R**<br>**rpsL.K88R**<br>**Mycobacterium_tuberculosis_16S.A1401G**<br>gyrB.V340L<br>thyA.T202A<br>kasA.G269S<br>folC.A420V<br>iniA.S501W<br>gidB.L79S	80.00%

OFX	**katG.S315T**<br>**gyrA.A90V**<br>**gyrA.D94G**	100.00%
**Nonredundant Union**	**embA.P913S**<br>**embB.D1024N**<br>**embB.D328G**<br>**embB.D328Y**<br>**embB.E378A**<br>**embB.G406A**<br>**embB.G406C**<br>**embB.G406D**<br>**embB.G406S**<br>**embB.M306I**<br>**embB.M306L**<br>**embB.M306V**<br>**embB.Q497K**<br>**embB.Q497R**<br>**embB.S297A**<br>**embB.Y319C**<br>**embB.Y334H**<br>**embC.N394D**<br>**embC.R738Q**<br>**embC.T270I**<br>**embC.V981L**<br>**embR.C110Y**<br>**ethA.A381P**<br>**ethA.D58A**<br>**gyrA.A90V**<br>**gyrA.D89G**<br>**gyrA.D89N**<br>**gyrA.D94A**<br>**gyrA.D94G**<br>**gyrA.D94N**<br>**gyrA.D94Y**<br>**gyrA.G88A**<br>**gyrA.G88C**<br>**gyrA.S91P**<br>**inhA.I194T**<br>**inhA.I21T**<br>**inhA.I21V**<br>**inhA.S94A**<br>**katG.G279D**<br>**katG.Q127P**<br>**katG.R463L**<br>**katG.S140N**<br>**katG.S315G**	78.81%

	**katG.S315I<br>katG.S315N<br>katG.S315T<br>katG.Y337C<br>ndh.R268H<br>ndh.V18A<br>pncA.A134V<br>pncA.A146T<br>pncA.C14R<br>pncA.D12A<br>pncA.D12N<br>pncA.D49A<br>pncA.F58L<br>pncA.G132S<br>pncA.G97S<br>pncA.H51Q<br>pncA.H57D<br>pncA.H82R<br>pncA.L159R<br>pncA.L172P<br>pncA.L27P<br>pncA.L4W<br>pncA.L85P<br>pncA.L85R<br>pncA.M175V<br>pncA.P54L<br>pncA.Q10P<br>pncA.Q10R<br>pncA.Q141P<br>pncA.R154G<br>pncA.S67P<br>pncA.T135P<br>pncA.T160P<br>pncA.T47A<br>pncA.T76P<br>pncA.V139A<br>pncA.V139L<br>pncA.V155G<br>pncA.V7G<br>pncA.V9G<br>pncA.W119R<br>pncA.W68G<br>pncA.W68R<br>pncA.Y103H<br>rpoB.S450L<br>rpsL.K43R**	

	**rpsL.K88Q**<br>**rpsL.K88R**<br>**Mycobacterium_tuberculosis_16S.A1401G**<br>**tlyA.N236K**<br>Mycobacterium_avium_23S.A2274G<br>folC.A420V<br>folC.I43A<br>folC.I43S<br>folC.I43T<br>folC.S150G<br>gidB.A134E<br>gidB.A138E<br>gidB.H48Y<br>gidB.L79S<br>gidB.P75S<br>gidB.S70R<br>gyrB.V340L<br>iniA.S501W<br>kasA.G269S<br>kasA.G312S<br>katG.A234G<br>katG.A431V<br>katG.G300W<br>katG.P131Q<br>rpoB.D516G<br>rpoB.H526T<br>rpoB.L511R<br>thyA.H207R<br>thyA.T202A	
